# Sexual and reproductive health knowledge and practices among youth with and without mental illness in Uganda: a comparative study

**DOI:** 10.1186/s41182-022-00444-1

**Published:** 2022-08-02

**Authors:** Emily Tumwakire, Scholastic Ashaba, Vincent Mubangizi, Yahaya Gavamukulya

**Affiliations:** 1 Department of Community Practice and Family Medicine, Faculty of Medicine, Mbarara University of Science and Technology, Mbarara, Uganda; 2 Ministry of Health, Uganda, Kampala, Uganda; 3 Department of Psychiatry, Faculty of Medicine, Mbarara University of Science and Technology, Mbarara, Uganda; 4 Department of Biochemistry and Molecular Biology, Faculty of Health Sciences, Busitema University, P.O. Box 1460, Mbale, Uganda

**Keywords:** Mental illness, Sexual and reproductive health, Youth, South Western Uganda

## Abstract

**Background:**

Sexual and reproductive health challenges among youth in low-income countries have persistently remained a public health challenge. In addition to these challenges, approximately 25% of youth experience a mental health illness, a situation anticipated to steeply increase especially in sub-Saharan Africa. However, there is still a scarcity of knowledge on the sexual and reproductive health of youth with mental illness in comparison to youth without mental illness in low-income countries. In this paper, the objective was to compare the sexual and reproductive health knowledge and practices among youth with mental illness and without mental illness at Mbarara Regional Referral Hospital (MRRH), South Western Uganda.

**Methods:**

Using a cross-sectional comparative study design, 104 youth with mental illness and 101 youth without mental illness were recruited as they sought medical health care services at MRRH. Structured interviews were conducted and they covered sexual and reproductive health knowledge and sexual practices.

**Results:**

205 youth were interviewed and of these 53 males and 51 females had mental illness while 49 males and 52 females did not have a mental illness. More youth without mental illness (61.7%) had more knowledge of sexual and reproductive health compared to youth with mental illness (38.3%) with a prevalence odds ratio of 0.29 (CI 0.16–0.52) and *p* value of 0.001. All youth were knowledgeable about contraceptive methods. Youth with MI engaged more in risky sexual practices though the difference wasn’t statistically significant.

**Conclusions:**

Youth generally have low sexual and reproductive health knowledge and this was found to be significantly lower in youth with mental illness compared to those without mental illness and they generally tend to engage in risky sexual behavior. It is recommended to incorporate SRH services among the mainstream general youth health care and mental health care services is critical to reducing sexual and reproductive health challenges among youth.

**Supplementary Information:**

The online version contains supplementary material available at 10.1186/s41182-022-00444-1.

## Introduction

Despite youth (15–24 years) being globally viewed as healthy and energetic beings, sexual and reproductive health (SRH) challenges continue to globally threaten their wellbeing [1]. Though a combined global improvement in the youth’s SRH has been globally noted, youth in low-income countries continue to experience significantly more SRH challenges, a situation whose global picture is anticipated to worsen by 2030 [1]. Youth especially in low-income countries continue to commonly experience a myriad of SRH problems and complications such as unintended pregnancies and unsafe abortions in addition to sexually transmitted infections (STIs), such as HIV, gonorrhea, syphilis, and sexual coercion [2]. Globally, 34% of all new HIV infections are recorded among youths of which 37% are from sub-Saharan Africa.

Approximately 75% of all mental illnesses (MI) start during the youthful years [3] and it is estimated that 25% of all youth at any one time are affected by MI [4]. Though the exact prevalence of MI among the youth in sub-Saharan Africa is unknown [5]. In Uganda, the prevalence of mental illness is estimated to be between 12 and 29% with anticipation of progressive increment over the years [6]. Mental illness among youth has been found to worsen their SRH challenges [7–9] and this can be attributed to the globally associated discrimination that significantly hinders seeking appropriate mental and SRH services [4]. In sub-Saharan Africa and Uganda, this is further worsened by a general lack of mental health care services [6, 10]. Mental illness also impairs an individual’s ability to control impulses and impairs judgment and cognition, an aspect that has been found to negatively influence their SRH more than those without mental illness [11, 12]. This negative influence on the SRH of people with mental illness is further evidenced by a globally higher prevalence of HIV among those with mental illness compared to the general population [13, 14].

More than half of youth generally engage in risky sexual behavior including early sexual debut, multiple sexual partners, not testing for HIV with sexual partners, sex trade, and no usage of contraceptives [15]. Despite early sexual debut being associated with engagement in risky sexual behavior [16, 17] a significant proportion of youth in Uganda are still having sexual debut at a very young age [18–20]. This sexual debut occurs when the majority significantly lack adequate SRH knowledge [19, 20]. A higher prevalence of risky sexual behaviors has been found among youth with MI compared to those without MI [8, 21–24]. In Uganda, depression among youth was found to be associated with having more sexual partners compared to youths without depression [7]. This higher prevalence of risky sexual behavior among youth with mental illness has been associated with higher rates of adolescent pregnancy [9, 24], abortions and unplanned pregnancies [25], and a higher prevalence of HIV compared to youth in the general population [13, 26–28].

Knowledge of SRH status of youth with mental illness in low-income countries is generally lacking with the majority of studies conducted among youth without mental illness. The objective of this study was to compare the sexual and reproductive health knowledge and practices of youth with and without mental illness in Mbarara Regional Referral Hospital, in South Western Uganda.

## Materials and methods

### Study design

The study was a cross-sectional comparative study involving youth with mental illness and youth without mental illness.

### Study setting

The study was conducted at Mbarara Regional Referral Hospital (MRRH) which serves the entire south western Uganda with a bed capacity of approximately 403. MRRH also serves as the teaching hospital for Mbarara University of Science and Technology and other neighboring medical institutes, such as Mayanja Memorial Training Institute and Bishop Stuart University. The hospital provides general medical care services in addition to specialist services. The study data for youth without mental illness was collected from general Out Patient Departments (OPD) which receive approximately 120 patients per day of which 30 are youths. The psychiatric outpatient clinic is conducted on Tuesdays and Wednesdays and approximately 100 patients are seen per clinic day of which, 30 are usually youths. Data for youth with MI was collected from this clinic.

### Study population

The study population comprised youth aged 15 to 24 years of age with mental illness and those without mental illness who were attending the mental health clinic and general OPD respectively at Mbarara Regional Referral Hospital.

### Sample size and sampling criteria

The sample size was estimated using Open Epi software (https://www.openepi.com/SampleSize/SSCohort.htm) using the Fleiss formula for cohort studies since the study involved two groups whose variables were compared.

It was earlier reported that 22.7% of youth aged 15 to 24 years in Uganda had risky sexual behavior [29]. This was set as a percentage of outcome in the unexposed group (youth without mental illness). In a study conducted among older youths at the university, it was found that the odds ratio of having multiple sexual partners in older youths with depression compared to those without depression was 2.5 [7] and this was set as the odds ratio in the exposed group. Setting the percentage of outcome in the unexposed group as 22.7%, the odds ratio as 2.5, a level of significance of 95%, and a power of 80%, gave a minimum total sample size of 180 participants. Catering for a non-response rate of 10%, a minimal sample size of 200 participants was considered. However, 205 participants were recruited including 104 participants with mental illness and 101 participants without mental illness.

### Eligibility criteria

#### Inclusion criteria

Youth aged 15–24 years of age with and without mental illness, with clarity on their mental status, who were fluent in English or Runyankore and were able to provide valid informed consent were eligible to participate.

#### Exclusion criteria

Youth with and without mental illness who were not in good physical and or mental health to be able to understand and withstand the interview process were also excluded. Youth with mental illness who had active symptoms as evidenced by mental state examination that hindered their ability to understand the contents of the consent form and that of the interview were also excluded from the study.

### Data collection and management

#### Study independent variable

Mental Illness, socio-demographic factors, such as age, education level, and environment.

#### Dependent variables

Risky sexual practices such as multiple sexual partners, having more than one concurrent sexual partner, non-use of contraceptives, inconsistent contraceptive use, sex purchase, having a one-night stand, and having unplanned sexual intercourse.

#### Data collection tools

The WHO recommended questionnaire for interview surveys with young people [30] was adapted and used for the study to assess their knowledge of puberty, contraception and contraceptive methods, STIs including HIV, and their sexual practices. The questionnaire was translated into Rukiga/Runyankore (Additional file 1) and used to collect data and back-translated into English (Additional file 2) and checked for consistency and accuracy.

Puberty knowledge was assessed and scored using six questions that asked about menstruation, wet dreams and common pregnancy myths such as a woman cannot get pregnant during the first time of having sex. HIV knowledge was assessed and scored using 12 questions which assessed if they knew about HIV, modes of transmission, how to prevent HIV transmission, and common myths about HIV/AIDS. STI Knowledge was assessed and scored using four questions that asked about STIs known, common signs and symptoms of STIs in men and women, and places where to get STI treatment from. Contraceptive knowledge was assessed by mentioning contraceptive methods known and knowing where to get the methods if needed.

Sexual practices were assessed by asking participants about being in a relationship, sexual activity, number of sexual partners, number of partners they have had sexual intercourse with, age at sexual debut, having concurrent partners, and use of contraceptives, such as condoms.

### Study procedure

A consecutive sampling technique was used to recruit the participants for interviews at MRRH outpatient clinics after obtaining the clinical health care services they came seeking. Recruitment was done consecutively until the predetermined sample size was reached. Youth without mental illness were enrolled from the general OPD where the attending clinicians conducted mental state exam to rule out mental illness before forwarding them to the study team for possible recruitment. The study team conducted another mental state exam to rule out mental illness prior to interviewing participants and to be able to put them in the group without mental illness. Youth with MI were enrolled from the mental health clinic as they sought their routine mental health care reviews and drug refills and mental status was done by the attending clinician to ensure they were mentally stable to comprehend the interview questions. These youth already had a mental illness diagnosis and were enrolled in the routine psychiatric outpatient clinic.

All participants were enrolled in the study after they provided written informed consent and assent for minors. All youth approached to participate in the study agreed to participate.

Mature minors (those below 18 years of age and use drugs or have an STI) and emancipated minors (those below 18 years and are pregnant, married, have a child, or cater for their livelihood) were allowed to provide written consent as per national research guidelines [31]. Those below 18 years and not falling under mature or emancipated minors provided written assent after the accompanying caretaker provided written informed consent.

Data was collected using questionnaires that were administered to the participants by the first author and 4 research assistants. Research assistants comprised 2 psychiatric nurses and 2 general nurses who had experience in interviewing youth and were fluent in both English and Rukiga/Runyankore. They were trained by the first author on how to use the study tools. The participants were assigned a male or female research assistant based on their preference.

### Data management and analysis

The data was cleaned and entered into Epi-Data after which it was exported to STATA 15 for analysis. All the collected data was included in the analysis because the questionnaires had complete data. All analyses were stratified by mental status using *t* test and Chi-square test. A *p* value of less than 0.05 was considered a significant difference between the two groups.

#### Bivariate analysis

All variables were categorized before bivariate analysis. Continuous variables were categorized for purposes of analysis to identify proportions with risky sexual behavior and those with not. Age at sexual debut was categorized into two groups: those who had a sexual debut at 15 years and below and those who had a sexual debut above 15 years. The number of partners was categorized into more than 2 and less than 2. A two-by-two table for each variable summarized the presence or absence of mental illness. The Chi-square test was used in variables, which had a proportion of more than 10 in both mental illness and no mental illness. Those with a proportion less than 10 were analyzed using Fischer’s test.

Wilcoxon’s rank sum/Mann–Whitney test were used to assess association in the continuous versus categorical data. *Z* scores with their respective *p* values were reported. Prevalence odds ratios were calculated to measure associations with mental illness.

To compare the sexual and reproductive health knowledge of youth with mental illness and youth without mental illness in Uganda. Answers to the questions were scored with 1 if correct and 0 if wrong and the totals were obtained. Grading that was utilized in UDHS 2016 was employed to categorize participants with less knowledge and more knowledge [20]. Participants who passed more than half (50%) of the assessment questions were classified as knowledgeable and those who scored less than half (50%) of the assessment questions were classified as being less knowledgeable.

To compare the sexual practices of youth with mental illness and youth without mental illness in Uganda. Proportions of youth engaging in various sexual practices were calculated and the respective prevalence odds ratios and *p* values were reported.

### Quality control

The study questionnaire was translated into local languages (Runyankore and Rukiga) and back-translated into English and checked for consistency and accuracy. The data was checked for completeness at the end of each day of data collection. Data collectors had experience in interviewing youth. The questionnaire was pretested before it was used in data collection.

## Results

### Socio-demographic characteristics of participants

Two hundred and five male and female participants of ages 15 years and 24 years were enrolled in the study. Youth with mental illness comprised 104 participants (53 males and 51 females) and those without mental illness comprised 101 participants (49 males and 52 females). The majority of the participants (47.7%) had attained secondary school. For those with mental illness, the majority (58.65%) had mood disorders while very few (9.6%) had substance use disorders (Table 1).

**Table 1:** Socio-demographic characteristics of participants

Variable	Overall	Mental illness	No mental illness	*p *value
	*N* = 205 (%)	*N* = 104 (%)	*N* = 101 (%)	
Gender				
Male	102 (49.76)	53 (50.96)	49 (48.51)	0.726
Female	103 (50.24)	51 (49.04)	52 (51.49)	
Age in years				
15–19	101 (49.27)	56 (53.85)	45 (44.55)	0.183
20–24	104 (50.73)	48 (46.15)	56 (55.45)	
Education level				
No education	7 (3.41)	4 (3.85)	3 (2.97)	0.960
Primary	52 (25.37)	25 (24.04)	27 (26.73)	
Secondary	98 (47.8)	50 (48.08)	48 (47.52)	
Tertiary	48 (23.41)	25 (24.04)	23 (22.77)	
Psychiatric conditions				
Psychosis		33 (31.73)		
Mood disorder	–	61 (58.65)		–
Substance abuse		10 (9.62)		

### Comparison of knowledge between youth with mental illness and those without mental illness

Overall youth without mental illness had more knowledge about sexual and reproductive health compared to youth with mental illness. Of the youth who passed more than 50% of all the knowledge questions, youth without mental illness comprised the biggest proportion (61.7%). (odds ratio (OR): 0.29 (CI 0.16–0.52) *p* value = 0.001). More youth without MI (71.4%) compared to those with MI (28.6%) scored above 50% in the puberty knowledge assessment. (OR: 0.32 (CI 0.15–0.71) a *p* value = 0.004) (Table 2).

**Table 2:** Details of sexual and reproductive health knowledge presented by mental health status

Knowledge scores	Overall *N* = 205	Mental illness, *N* = 104	No mental illness, *N* = 101	Adjusted OR (95% CI), *p* values
Puberty				
Less knowledge	170	94 (55.3)	76 (44.7)	OR 0.32 (0.15, 0.71) *p* = 0.004*
More knowledge	35	10 (28.6)	25 (71.4)	
HIV				
Less knowledge	120	66 (55.0)	54 (45.0)	OR 0.66 (0.38, 1.15), *p* = 0.146
More knowledge	85	38 (45.0)	47 (55.0)	
STIs				
Less knowledge	63	47 (74.6)	16 (25.4)	OR 0.23 (0.11, 0.44) *p* = 0.015*
More knowledge	142	57 (40.1)	85 (59.9)	
Overall knowledge				
Less knowledge	85	58 (68.2)	27 (31.8)	OR 0.29 (0.16, 0.52) *p* = 0.001*
More knowledge	120	46 (38.3)	74 (61.7)	

^*^Statistically significant value

**Fig. 1:**
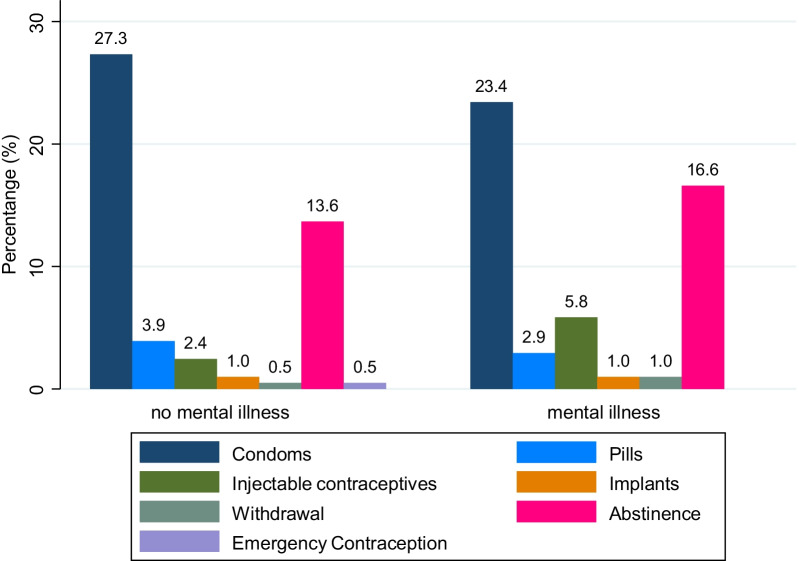
Contraceptive methods mentioned by youth with mental illness and youth without mental illness and sex

All participants knew at least one contraceptive method. Condoms were the most commonly known contraceptive method. However, for females with mental illness, abstinence was the most commonly mentioned method followed by condoms (Fig. 1).

### Comparison of sexual practices between youth with mental illness and those without mental illness

Generally, 156 (76.1%) of the total participants were in a relationship, and of these, 56.6% had ever engaged in sexual intercourse. More youth without MI (51.28%) were in a relationship. Of those in a relationship, youth with MI comprised a bigger proportion (52.6%) compared to those without MI (47.4%), who have ever had sexual intercourse. More youth without MI adopt safer sexual practices with a slightly higher proportion (52.7%) compared to those with MI (47.3%) who reported condom use during their sexual intercourse. There were no statistically significant differences in sexual practices of youth with mental illness and those without mental illness (Table 3).

**Table 3:** Sexual practices presented for all participants and by mental health status

Sexual practices	Overall	Mental illness (%)	No mental illness (%)	Adjusted odds ratios (95% CI), *p* value
In a relationship	156	76 (48.72%)	80 (51.28%)	OR 0.71 (0.37–1.35) *p* = 0.309
Ever had sex	115	60 (52.6%)	55 (47.4%)	OR 1.70 (0.83–3.50) *p* = 0.575
Unplanned sex	39	21 (53.8%)	18 (46.15%)	OR 0.90 (0.42–1.95) *p* = 0.797
Causal relationship	58	33 (56.9%)	25 (43.1%)	OR 1.69 (0.93–2.45) *p* = 0.291
Concurrent partners	53	24 (45.3%)	29 (54.7%)	OR 0.81 (0.42–1.57) *p* = 0.538
> 2 lifetime partners	75	36 (48%)	39 (52%)	OR 0.95 (0.38–1.53) *p* = 0.863
Sexual debut at 15 years and below	23	14 (60.9%)	9 (39.1%)	OR 1.56 (0.3–2.82) *p* = 0.398
Had a one-night stand	32	17 (53.1%)	15 (46.9%)	OR 1.05 (0.28–1.815) *p* = 0.768
Sex purchase	23	12 (56.5%)	10 (43.5%)	OR 1.3 (0.55–3.06) *p* = 0.556
Use of condoms	93	44 (47.3%)	49 (52.7%)	OR 0.78 (0.45–1.35) *p* = 0.372
Consistent contraceptive use	16	8 (50.0%)	8 (50.0%)	OR 0.78 (0.20–3.04) *p* = 0.812
Know HIV status	159	79 (49.7%)	80 (50.3%)	OR 0.83 (0.38–1.28) *p* = 0.578
Incorrect measures to reduce HIV	70	37 (52.9%)	33 (47.1%)	OR 1.07 (0.62–1.52) *p* = 0.507

## Discussion

The major aim of this study was to compare the sexual and reproductive knowledge and practices of youth with and without mental illness and to establish if a difference exists between these two groups. Most youths had poor sexual and reproductive knowledge and this was worse during puberty. They had a fair knowledge of HIV and STIs. This limited SRH knowledge is discussed below in comparison to other previously conducted studies among youths generally. Though there were no significant differences identified in the sexual practices, youth generally engaged in risky sexual practices, such as early sexual debut, sex trade, concurrent and multiple partners, and these are discussed below in comparison to other previously conducted.

The study findings support the hypothesis that there is indeed a difference in SRH knowledge between youth with mental illness ad those without mental illness. Youth with mental illness generally had low SRH knowledge compared to youth without MI and this difference was statistically significant. However, comparable studies that assessed and compared SRH knowledge in these two groups were not found. Despite youth without MI having more SRH knowledge compared to those with MI, both groups, in general, had low knowledge with only 58.5% of all the participants getting the assessment questions correct. This finding is similar to that which found that Ugandan youth generally had low SRH knowledge [19]. For instance, they found that only half of the youth had good knowledge about STIs which was assessed similarly to our study by asking about known STIs, signs and symptoms of STIs in men and women, and knowing where to get medical attention in case they have an STI. However, another study conducted in Uganda reported that 85.6% of the assessed youth had good STI knowledge [29]. This is significantly different from our study which shows that 69.3% of the assessed youth have good STI knowledge. This difference could be attributed to the difference in assessment whereby Palomino, Kadengye, and Mayega [29] only assessed being able to mention one STI yet our study and that of Crossland et al. [19] did a more comprehensive STI assessment with more questions asking for a correct STI, signs and symptoms of STI in both men and women and knowing places from where STI treatment can be obtained.

Though the study did not find any statistically significant difference in HIV knowledge among youth with mental illness and those without, more youth had less knowledge about HIV with 58.5% scoring below 50% of the assessed questions in HIV knowledge. These findings are similar to those of the 2016 UDHS that assessed HIV knowledge similarly to our study with 52.8% of female youth and 44.8% of male youth having good comprehensive HIV knowledge [20].

All participants in the study were found to know at least one contraceptive method with condoms being the most cited modern contraceptive method. This finding was consistent with the 2016 UDHS findings which showed that almost 99% of assessed youths knew about modern methods of contraceptives and condoms were the most known modern contraceptive [20]. Similar findings were also found among Kenyan youths [32].

The majority of the participants (83%) were found to have low knowledge about puberty which was assessed with questions on menstruation, wet dreams, and pregnancy myths. Although similar studies assessing puberty knowledge among youths aged 15–24 years are sparse, Kemigisha et al. [18] conducted an almost similar assessment among young adolescents aged 10–14 years in south western Uganda and found that 72.1% of the young adolescents could not correctly answer more than 50% of the assessment questions. This shows that generally, youth in Uganda have low knowledge about puberty.

Our study findings show that there were no statistically significant differences in sexual practices of youth with MI and that of youth without MI and therefore rejected our study hypothesis that youth with MI engage more in risky sexual practices compared to youth without MI. Our study assessed being sexually active, having unplanned sexual intercourse, having casual relationships, having concurrent sexual partners, having more than 2-lifetime partners, age at sexual debut, having one-night stands, involvement in the sex trade, and consistent use of condoms with sexual partners. Though the proportions of youth with MI who engaged in these risky sexual practices were consistently slightly more, the differences were not statistically significant. These findings were similar to those that reported that the age of sexual debut among youth with first-time psychosis and those without psychosis be 16.3 years and 16.6 years respectively [33]. Similar findings were found in the USA [34]. These two study findings on sexual debut are similar to our study findings which showed that sexual debut occurred before 19 years of age irrespective of mental status and location.

Our study found that of the 23 participants who engaged in sex purchase, 56.5% had MI while 43.5% did not have MI, a difference that was not statistically significant. However, Brown, Lubman, and Paxton [33] reported a statistically significant difference in sex purchase between youth with MI and youth without MI. In their study, 73% of those who engaged in sex purchase had MI compared to 27% who did not have MI.

Though our findings did not show significant differences in sexual practices of youth with and without MI, similar comparative studies conducted among adults with MI and those without MI show significant differences in sexual practices between the two groups [27, 35, 36]. For instance, it was reported that women with MI had significantly more lifetime partners, more concurrent partners, and less condom use compared to women who did not have MI. A study conducted among 18–49-year-old Ugandans with and without MI found significant differences in consistent condom use and the number of lifetime partners with people with MI using condoms less and having more partners compared to those without MI [28].

Our study was facility-based and the participants were not randomly selected. The participants might therefore not be representative of the youth population in South Western, Uganda. The youth recruited might be those with good health-seeking behavior and those with mental illness who are adherent to their medications and therefore mentally stable and able to live a normal life. Furthermore, the study relied on self-reports from participants with some required information especially on the sexual practices being retrospective. The information collected on sexual practices might therefore have been affected by the recall and social desirability bias. However, privacy and confidentiality were ensured to encourage participants to freely share the information.

## Conclusion

Youth generally have low sexual and reproductive health knowledge and this was found to be significantly lower in youth with mental illness compared to those without mental illness. Youth with mental illness and those without mental illness similarly engage in risky sexual practices and they, therefore, need more preventive and promotive health education on sexual and reproductive health. It is recommended that youth be given sexual and reproductive health education using various avenues such as incorporating sexual and reproductive health education in all youth health care and mainstream mental health care services to maximize all opportunities to give them more knowledge needed in decision making and adoption of safe sexual practices. Similarly, more comparative and prospective studies among people with mental illness and those without mental illness need to be conducted in low-income countries to obtain a clearer picture of their sexual and reproductive health and identify the needs that might be different from the general population.

## Supplementary Information

**Additional file 1. **Runyankore Questionnaire.

**Additional file 2.** English Questionnaire.

## Data Availability

Raw data can be obtained from the corresponding author upon reasonable request.

## Abbreviations

ADHD: Attention Deficit Hyperactivity Disorder
AIDS: Acquired immunodeficiency syndrome
CDC: Centres for Disease Control
FRC: Faculty Research Committee
HIV: Human immunodeficiency virus
MI: Mental illness
MRRH: Mbarara Regional Referral Hospital
MUST: Mbarara University of Science and Technology
OPD: Out Patient Department
OR: Odds ratio
REC: Research Ethics Committee
SRH: Sexual and Reproductive Health
STI: Sexually transmitted infection
UBOS: Uganda Bureau of Statistics
UDHS: Uganda Demographic Health Survey
UNAIDS: Joint United Nations Programme on HIV and AIDS
UNESCO: United Nations Educational, Scientific and Cultural Organization.
UNFPA: United Nations Population Fund
WHO: World Health Organization

## References

[1] World Health Organization (2018). Adolescent pregnancy; key facts.

[2] Chandra-Mouli V, Camacho AV, Michaud PA (2013). WHO guidelines on preventing early pregnancy and poor reproductive outcomes among adolescents in developing countries. J Adolesc Health.

[3] Kessler RC, Berglund P, Demler O, Jin R, Merikangas KR, Walters EE (2005). Lifetime prevalence and age-of-onset distributions of DSM-IV disorders in the national comorbidity survey replication. Arch Gen Psychiatry.

[4] United Nations (2014). Mental Health Matters: social inclusion of youth with mental health conditions.

[5] Charlson FJ, Diminic S, Lund C, Degenhardt L, Whiteford HA (2014). Mental and substance use disorders in Sub-Saharan Africa: predictions of epidemiological changes and mental health workforce requirements for the next 40 years. PLoS ONE.

[6] Ministry of Health (2017). Child and Adolescent Mental Health policy guidelines.

[7] Agardh A, Elizabeth CG, Östergren PO (2012). Youth, sexual risk-taking behavior, and mental health: a study of university students in Uganda. Int J Behav Med.

[8] Chen MH, Wei HT, Bai YM (2019). Sexually transmitted infection among adolescents and young adults with bipolar disorder: a nationwide longitudinal study. J Clin Psychiatry.

[9] Vigod SN, Dennis CL, Kurdyak PA, Cairney J, Guttmann A, Taylor VH (2014). Fertility rate trends among adolescent girls with major mental illness: a population-based study. Pediatrics.

[10] Okasha A (2002). Mental health in Africa: the role of the WPA. World Psychiatry.

[11] Raja M, Azzoni A (2003). Sexual behavior and sexual problems among patients with severe chronic psychoses. Eur Psychiatry.

[12] Deckman T, Nathan DeWall C (2011). Negative urgency and risky sexual behaviors: a clarification of the relationship between impulsivity and risky sexual behavior. Pers Individ Dif.

[13] Hughes E, Bassi S, Gilbody S, Bland M, Martin F (2016). Prevalence of HIV, hepatitis B, and hepatitis C in people with severe mental illness: a systematic review and meta-analysis. Lancet Psychiatry.

[14] Lundberg P, Nakasujja N, Musisi S, Thorson AE, Cantor-Graae E, Allebeck P (2013). HIV prevalence in persons with severe mental illness in Uganda: a cross-sectional hospital-based study. Int J Ment Health Syst.

[15] CDC. Sexual risk behaviors can lead to HIV, STDs, & teen pregnancy. 2019.

[16] Mbalinda SN, Kiwanuka N, Eriksson LE, Wanyenze RK, Kaye DK (2015). Correlates of ever had sex among perinatally HIV-infected adolescents in Uganda. Reprod Health.

[17] Mmari KN, Kaggwa E, Wagman J, Gray R, Wawer M, Nalugoda F (2013). Risk and protective correlates of young women’s first sexual experiences in Rakai, Uganda. Int Perspect Sex Reprod Health.

[18] Kemigisha E, Bruce K, Nyakato VN, Ruzaaza GN, Ninsiima AB, Mlahagwa W, Leye E, Coene G, Michielsen K (2018). Sexual health of very young adolescents in South Western Uganda: a cross-sectional assessment of sexual knowledge and behavior. Reprod Health.

[19] Crossland N, Hadden WC, Vargas WE, Valadez JJ, Jeffery C (2015). Sexual and reproductive health among Ugandan youth: 2003–04 to 2012. J Adolesc Health.

[20] UBOS. Uganda 2016 demographic and health survey: key findings. Uganda 2016 Demogr. Health Surv. 2016.

[21] Flory K, Molina BSG, Pelham WE, Gnagy E, Smith B (2006). Childhood ADHD predicts risky sexual behavior in young adulthood. J Clin Child Adolesc Psychol.

[22] Ramos Olazagasti MA, Klein RG, Mannuzza S, Belsky ER, Hutchison JA, Lashua-Shriftman EC, Xavier Castellanos F (2013). Does childhood attention-deficit/hyperactivity disorder predict risk-taking and medical illnesses in adulthood?. J Am Acad Child Adolesc Psychiatry.

[23] Barkley RA, Fischer M, Smallish L, Fletcher K (2006). Young adult outcome of hyperactive children: adaptive functioning in major life activities. J Am Acad Child Adolesc Psychiatry.

[24] Østergaard SD, Dalsgaard S, Faraone SV, Munk-Olsen T, Laursen TM (2017). Teenage parenthood and birth rates for individuals with and without attention-deficit/hyperactivity disorder: a nationwide cohort study. J Am Acad Child Adolesc Psychiatry.

[25] Marinelli M, Lorenzo LS, Zaratiegui R. Reproductive characteristics of female outpatients of childbearing age with affective disorders. In: Bipolar disorder. Malden: Wiley-Blackwell; 2012. p. 102–3.

[26] Bauer-Staeb C, Jörgensen L, Lewis G, Dalman C, Osborn DPJ, Hayes JF (2017). Prevalence and risk factors for HIV, hepatitis B, and hepatitis C in people with severe mental illness: a total population study of Sweden. Lancet Psychiatry.

[27] Lundberg P, Nakasujja N, Musisi S, Thorson AE, Cantor-Graae E, Allebeck P (2015). Sexual risk behavior, sexual violence, and HIV in persons with severe mental illness in uganda: hospital-based cross-sectional study and national comparison data. Am J Public Health.

[28] Lundberg P, Rukundo G, Ashaba S, Thorson A, Allebeck P, Stergren PO, Cantor-Graae E (2011). Poor mental health and sexual risk behaviours in Uganda: a cross-sectional population-based study. BMC Public Health.

[29] Palomino González R, Kadengye DT, Mayega RW (2019). The knowledge-risk-behaviour continuum among young Ugandans: what it tells us about SRH/HIV integration. BMC Public Health.

[30] Cleland J, Ingham R, Stone N (2001). Asking Young people about sexual and reproductive behaviour: illustrative questionnaire for interview.

[31] UNCST (2014). National guidelines for research involving humans as research participants.

[32] Mbugua SM, Karonjo JM (2018). Reproductive health knowledge among college students in Kenya. BMC Public Health.

[33] Brown A, Lubman DI, Paxton S (2010). Sexual risk behaviour in young people with first episode psychosis. Early Interv Psychiatry.

[34] Dickerson F, Brown C, Kreyenbuhl J, Goldberg R, Juan L, Dixon L (2004). Sexual and reproductive behaviors among persons with mental illness. Psychiatr Serv.

[35] Dinc H, Boyacioglu NE, Ozcan NK, Enginkaya S (2019). Reproductive and sexual health in women with bipolar disorder: a comparative study. Dusunen Adam.

[36] Marengo E, Martino DJ, Igoa A, Fassi G, Scápola M, Urtueta M, Strejilevich SA (2015). Sexual risk behaviors among women with bipolar disorder. Psychiatry Res.

