# Supplementary material for: Sexual and reproductive health knowledge and practices among youth with and without mental illness in Uganda: a comparative study
Source: Trop Med Health. 2022 Aug 2;50:51. doi: 10.1186/s41182-022-00444-1 (PMC9344651; doi:10.1186/s41182-022-00444-1)
Supplement: Supplementary file 1 — Additional file 1. Runyankore Questionnaire. [file 41182_2022_444_MOESM1_ESM.docx]

**Runyankore Questionnaire**

**Ekicweka kya 1**: Ebiri kukukwataho nana kworikuhikana n’abantu hamwe n’entasya Socioeconomic and Demographic characteristics

1. Obuhangwa bw’owayetaba omu okucondoza

|  | Omushaija/omutsigazi |  | Omwishiki/omukazi |
| --- | --- | --- | --- |

1. Emyaka y’owayetaba omu kucondoza ……………………………..
2. Okashoma wahika omu kyakangahi?

|  | Tindashomire/Tahariho |  | Purimare |  | Siniya |  | Tekiniko/kolegi |
| --- | --- | --- | --- | --- | --- | --- | --- |

**Ekicweka kya 2:** ahi ori kwiha okumanyisibwa nan’obwengye ahaby’amagara ebikwateirine nan’oruzaro/eby’okuzara/okwegaita.

1. Notunga ota okumanyisibwa haby’omushogoyo? kuruga nkahi(egyero eyi emibiri yabojo n’abaishiki eri kuhinduka baba bari omu myaka y’okunyeta/y’obunyeto kandi ni bukomoko ki obu ori kwesiga ninga obwori kuteekateeka ngu nibwo buhikire kimwe?) (gorora ekisharamo kimwe ahabya hebwoyo habwa a, b hamwe na c)

| ekyokusharamu | 1. Obukomoko | 1. Ekirikukirayo preferred | 1. Obuhikire kimwe |
| --- | --- | --- | --- |
| Ah’ishomero |  |  |  |
| Zamagazinis hamwe n’amahurire |  |  |  |
| Emizano/zafirimu |  |  |  |
| Radiyo |  |  |  |
| Ahamikutu yokuhikanisa abantu (facebook, watsapp) |  |  |  |
| Abashaho |  |  |  |
| Emikago/abanywani |  |  |  |
| Abazaire |  |  |  |
| Abinkuzarwa nabo |  |  |  |
| Ahandi(handika omuhanda ogu waha) |  |  |  |

1. Omwishiki kutandika kuza omumicwe/omukwezi nikimanyisa ki ahar’iwe/nokikyenga ota?

……………………………………………………………………………………………

1. Omwojo okunyama akarota haza akashohoza amaizi g’ekishaija atakimanyise nikimanyisa ki ahar’iwe?

……………………………………………………………………………………………

1. Omukazi nabasa kutwara enda omurundi gwe gwokubaza kwegaita/kuterana n’omushaija.

|  | Namazima |  | Timazima |  | Tinkumanya/tinkineho buhame |
| --- | --- | --- | --- | --- | --- |

1. Omukazi narekyeraho kukura yaheza kuterena/kwegaita n’omushaija omurundi gwokubanza.

|  | Namazima |  | Timazima |  | Ttikumanya/Tinkineho buhame |
| --- | --- | --- | --- | --- | --- |

1. Kuzanisa ebicweka byawe by’ekihama kweshemeza kine akabi kahahango ah’amagara.

|  | Namazima |  | Timazima |  | Ttikumanya/tinkineho buhame |
| --- | --- | --- | --- | --- | --- |

1. Omukazi nabasa kutwara enda yayegaita/yaterana n’omushaija rwagati yo kwezi kwe micwe.

|  | Namazima |  | Timazima |  | Tikumanya/tinkineho buhame |
| --- | --- | --- | --- | --- | --- |

**Ekicweka kya 3:** Okumanya hamwe n’enkozesa y’emiringo y’embaririra yaruzaro

1. Emiringo y’okubarira oruzaro ey’orikmanya neha(tikinga eyagambwaho)

|  | kodomu/obupira |  | Obujuma |  | Ekikato |  | obwomumukono |  | Omushaija kumarira aheru |  | Obutegaita n’omushaija |  | Ogwo kwekigira enda waheza  kwegaita oterinzire |
| --- | --- | --- | --- | --- | --- | --- | --- | --- | --- | --- | --- | --- | --- |

1. nat does this mean??What options are you referring to?Notekateka ngu emuringo eshemerire eminyeto neha

|  | kodomu/obupira |  | Obujuma |  | Ekikato |  | obwomumukono |  | Omushaija kumarira aheru |  | Obutegaita n’omushaija |  | Ogwo kwekigira enda waheza  kwegaita oterinzire |
| --- | --- | --- | --- | --- | --- | --- | --- | --- | --- | --- | --- | --- | --- |

1. Nomanya aho kwiha emiringo eyagambwaho haruguru?

|  | Ego |  | Ngaha |
| --- | --- | --- | --- |

1. Yaba ego, nemyanya eha eyorikumany?(tikinga emiringo eya hebwa)

|  | Omw’irwariro rya gavumenta |  | Omw’irwariro ry’omuntu buntu |  | Eduuka y’emibazi |  | Supamaketi/eduuka |  | Ahi eminyeto eri kwerundanira. |
| --- | --- | --- | --- | --- | --- | --- | --- | --- | --- |

**Ekicweka kya 4 Eki ori kumanya aha kakoko kasirimu/sirimu hamwe n’endwara z’obushambani.**

1. Warahurireho aha kakoko ka sirimu ninga sirimu (*use local terms*)?

|  | ego |  | ngaha |
| --- | --- | --- | --- |

1. Omuntu natunga ata akakoko ka sirimu?

………………………………………………………………………………………….

1. Hati naza kukushomera bimwe habikwatairine n’akakoko ka sirimu/sirimu. Ngambira yaba namazima, ninga timazima ninga torikumanya
2. Nikibasika kukiza sirimu

|  | Namazima |  | Timazima |  | Tinkumanya/tikineho buhame |
| --- | --- | --- | --- | --- | --- |

1. Nimbasa kureba omuntu ngambe yaba aine akakoko kasirimu/Nimbasa kumanya omuntu yaba ayine akakoko kasirimu namureba bureba.

|  | Anamazima |  | Timazima |  | Tikumanya/tikineho buhame |
| --- | --- | --- | --- | --- | --- |

1. Omuntu aine akakoko kasirimu naba ahweire amagufa ninga naba aine amagara mabi omumuringo gutari gumwe.

|  | Namazima |  | Timazima |  | Tikumanya/tikineho buhame |
| --- | --- | --- | --- | --- | --- |

1. Abantu nibabasa kuceberwa omumuringo gworobi/gwanguhi munonga kumanya yaba baine akakoko kasirimu

|  | Namazima |  | Timazima |  | Tikumanya/tikineho buhame |
| --- | --- | --- | --- | --- | --- |

1. Ningyero ki eyi omunyeto gukubasa kwekumamo bbutatunga akakoko kasirimu.

|  | Kukozesa kondomu |  | Kugira omukundwa omwe omwesigwa |  | Obutashambana/obutegaita |
| --- | --- | --- | --- | --- | --- |

1. Wara yekyebizeho akakoko kasirimu?

|  | ego |  | Ngaha |
| --- | --- | --- | --- |

1. Noteekateeka ngu nikikuru kwekyebeza akakoko kasirimu

|  | Ego |  | Ngaha |  |  |
| --- | --- | --- | --- | --- | --- |

1. Notekateka nikikuru kumanya omukundwa wawe kwayemerire omubya kakoko kasirimu?

|  | Ego |  | Ngaha |  |  |
| --- | --- | --- | --- | --- | --- |

1. . Oihireho akakoko kasirimu/sirimu warahurireho endijo ndwara ei abashaija ninga abakazi bakukwatwa ahanyima yokushambana?

|  | Ego |  | Ngaha ( guruka oze 25) |
| --- | --- | --- | --- |

1. Yaba ego, gamba ezo ezorikumanya?

………………………………………………………………………………………………

1. Obubonero bw’endwara z’obushambani omubashaija nibuha?

…………………………………………………………………………………………………

1. Obubonero bw’endwara z’obushambani omu bakazi nibuha?

………………………………………………………………………………………………….

1. Munywani wawe kuyakuba nayetaga obujanjabi bw’endwara y’obushambani, nabwiha hi?

………………………………………………………………………………………………………

.

**Ekicweka kya 5:** okuza omumubonano/okwegaita obwahati/obu herurukire

1. Waragizireho omukundwa/omurigirwa? Omukundwa/omurigirwa nimanyisa omwojo/omwishiki ouwahurire wakunda munonga ninga kwenda kwegaita nawe kandi ouwagyenzire nawe awutu/kutambaramu hataine muntu mukuru owumugyenzire nawe?.

|  | Ego |  | Ngaha( Guruka oze kicweka 6) |
| --- | --- | --- | --- |

1. Ogizire abakundwa/abarigirwa bangahi?...............................(yaba omwe, guruka kicweka 7)

Buza ebibuzo ebyakurataho bikwatiraine aha bwahati(omwahihi/omubiro bitari byahare) omukundwa/omurigirwa(mwete “omuntu ogwe”)

1. Omuntu ogwe aine emyaka engahi? Buuza emyaka ye hati? …………………………..
2. Omubwire obwo obu wabire ori/obu obire omuri rukundo n’omuntu ogwe, obire wagizire rukundo nomuntu ondijo?

|  | Ego |  | Ngaha |
| --- | --- | --- | --- |

1. Nobasa kushoborore ota omukago gwawe nogwo muntu? Gukaba (nogwo)

|  | Omunywani bunywani/omunywani kyonka. |
| --- | --- |
|  | Nomukago guhami konka gutaine ekigyendererwa kyo kushwerana/Okutasya. |
|  | Omukago mukuru gukubasa kuhendera omubushwere/okutasya. |

1. Eiwe nogwo muntu mukatunga okukwatana kwe mibiri yanyu, tugire nga okwekwata omugaro, okwefumbata omukifuba/okwegwa omunnda ninga okwenywegyera.

|  | Ego |  | Ngaha |
| --- | --- | --- | --- |

1. Okanywegyeraho ogwe muntu aha minywa?

|  | Ego |  | Ngaha |
| --- | --- | --- | --- |

1. Eiwe n’ogwo muntu mwarayekwasireho omubicweka by’ekihama?

|  | Ego |  | Ngaha |
| --- | --- | --- | --- |

1. Eiwe nogwo muntu mwara yegaisireho?

|  | Ego |  | Ngaha (guruka oze kicweka 6) |
| --- | --- | --- | --- |

**Ekicweka kyabo abarashambaineho/abarayegaisireho.**

1. Teekateeka enyimaho obu wayegaita nomukundwa wawe owoyine obwahati omurundi gwokubanza, nogira ngu. SHOMA EBI.

|  | Nkagyema omuntu ogwe kwegaita atarikwenda |
| --- | --- |
|  | Nkabihabiha omuntu ogwe kwegaita |
|  | Omuntu ogwe akambihabiha kwegaita |
|  | Omuntu ogwwe akagyema kwegaita |
|  | Twena/twembiri tukaba nitukyenda |

1. Kandi nobasa kugira ngu kikaba kiteebeekanisibwe ninga kitateekateekirwe?

|  | Kitebekanisibwe |  | Kitateekateekirwe |
| --- | --- | --- | --- |

1. Ogu nigwo gwabire guri omurundi gwawe gwokubanza kwegaita kuhika aha mbugiro omugara gawe?

|  | Ego |  | Ngaha |
| --- | --- | --- | --- |

1. Okaba oine emyaka engahi obu wayegaita n’omuntu ogwe omurunudi gwokubanza?.....................................
2. Aha murundi gw’okubanza ogwe, eiwe ninga ogwe muntu haine ayakozire ekintu kyona kukingira/kwerinda kutwara enda?

|  | Ego |  | Ngaha (guruka oze 43) |
| --- | --- | --- | --- |

1. Yaba ego, nomuringo guha ogu wakozise?.....................................................................

|  | Kondomu |  | Obujauma |  | Ekikatu |  | Obwahamukono |  | Omushaijakumariraaheru |  | Obutegaita |  | Ogwo kwekigira enda waheza  kwegaita oterinzire |
| --- | --- | --- | --- | --- | --- | --- | --- | --- | --- | --- | --- | --- | --- |

1. Waraganiraho aha by’okubaririra oruzaro n’omuntu ogwe? Yaba ego okaganira aha by’okubaririra oruzaro mutakegisire ninga mwaherize kwegaita omurundi gw,okubanza?

|  | Tindakiganireho |  | Tutakegaisire |  | Hanyuma y’okwegaita. |
| --- | --- | --- | --- | --- | --- |

1. Oihireho omurundi gwokubanza, eiwe nan’ogwe muntu mwara koziseho omuringo ogundi kwerinda kutwara enda?

|  | Ego burikaire |  | Ego obumwe |  | Tikikabahoga (guruka oze 46) |
| --- | --- | --- | --- | --- | --- |

1. Eiwe n’omuntu ogwe nimukira kukozesa muringo ki?

|  | Kondomu/obupira |  | Obujuma |  | Ekikatu |  | Obwahamukono |  | Omushaija kamarira aheru |  | Obutagaita |  | Ogwo kwekigira enda waheza  kwegaita oterinzire/otekumire |
| --- | --- | --- | --- | --- | --- | --- | --- | --- | --- | --- | --- | --- | --- |

1. Wara gizire okukwatwaho kugira ngu nobasa kukwatwa sirimu ninga endijo endwara y’obushambani kuruga aha muntu ogwe? Yaba ego, okakwatwoho ekirikwingana ki?

|  | Nkakwatwaho munonga |  | nkakwataho kukye |  | Tidakwatsirweho (guruka oze kicweka 6) |
| --- | --- | --- | --- | --- | --- |

1. Okabasa kugira eki wakora kucendeza aha migisha y’okukwatwa endwara?

|  | Ego |  | Ngaha |
| --- | --- | --- | --- |

1. Yaba eri ego, okakora ki? Buririza?…………………………………………………………………………..

**Ekicweka 6:** ebika by’okwegaita nabakazi/abashaija.

*Opening statement if participant had not had a girl/boyfriend before:*

Waba wangambire ngu tokagira omurigirwa/omukundwa. Hati ninyenda kukubuza aha miriingo yokwegaita yona eyi wara rabiremu.

*Opening statement for participants with or have had a boy/girlfriend before:*

Waba wangambire akakwate kwawe n’omuntu ogwe. Oihireeho we hamwe nomukundwa wena eyabandize, hati nenyenda kukubuza aha bika bya bakundwa abandi abu wakubasa kuba warabiremu.

1. Eminyeto emwe/abana bato nibegaita rumwe na rumwe batakimanyise/bata kyetebekanisise, katugire hanyuma y’embaga ninga baheza kunywamu. Eki kyarakubireho?

|  | Ego |  | Ngaha (guruka oze 58) |
| --- | --- | --- | --- |

1. Oyegaisire emirundi engahi erya rimwe na rimwe otakyetebekanisise?..................................
2. Eiwe ninga omukundwa wawe akakozesa omuringo gwona gw’okubariri oruzaro obumwayegaita muteeteebekanise?

|  | Buri kaire |  | Obumwe |  | Tikirabireho **(skip 52**) |
| --- | --- | --- | --- | --- | --- |

1. Yaba buri kaire ninga obumwe, nimuringo ki ogu mwakozise?

|  | Kondomu/obupira |  | Obujuma |  | Ekikato |  | Akahamukono |  | Omushaija kumarira aheru |  | Obutegaita |  | Ogwo kwekigira enda waheza  kwegaita oterinzire |
| --- | --- | --- | --- | --- | --- | --- | --- | --- | --- | --- | --- | --- | --- |

1. Eminyeto emwe/abana bato nibashashura sente ninga ebihembo kwenda kwegaita?eki kyarakubireho?

|  | Ego |  | Ngaha |
| --- | --- | --- | --- |

1. Eminyeto emwe/abana nibakira/nihebwa esente nninga ebihembo kwenda kwegaita. Eki kyarakubireho?

|  | Ego |  | Ngaha |
| --- | --- | --- | --- |

1. Yaba ego, n’abashaija/abakazi bangahi abu oyegaisire nabo habwe sente ninga ebihembo? …………………………………………………………………………..
2. Eiwe ninga ouwayegaisire nawe haine eyakozire ekintu kyona kwenda kwerinda enda obwire bwe?

|  | Buri kaire |  | Obumwe |  | Tikirabireho |
| --- | --- | --- | --- | --- | --- |

1. Yaba buri kaire ninga obumwe, nimuringo ki gw’embaariri yaruzaro ogu mwakozise?

|  | Kondomu/obupira |  | Obujuma |  | Ekikato |  | Akahamukukono |  | Omushaija kumarira aheru |  | Obutegaita |  | Ogwo kwekigira enda waheza  kwegaita oterinzire |
| --- | --- | --- | --- | --- | --- | --- | --- | --- | --- | --- | --- | --- | --- |

1. Omu magara gawe gona oyegaisire nabantu bangahi? …......................................
2. NEBY’ABO ABATAKEGAITAGA

| 59 | Abantu nibabasa kugira enshonga zitarikushana habwenki batarikwegaita. Ninza kukushomera zimwe aha shonga. Ninshaba ongambire buri hashonga yaba nekukwataho ninga terikukwataho. | Ninyikirizana nayo | Tikwikirizana nayo | Tindikumany/tikineho buhame. |
| --- | --- | --- | --- | --- |
| 1 | Nimpurira ntaketeeketeekire kwegaita. |  |  |  |
| 2 | Tinkatungire mugisha |  |  |  |
| 3 | Niteekateeka ngu akwegaita ntankashwire kigwire. |  |  |  |
| 4 | Nintina kutwara enda /okutweka |  |  |  |
| 5 | Nintina kukwatwa akakoko kasirimu/sirimu ninga ezinda endwar z’obushambani. |  |  |  |

1. Kandi hati nyine ekibuzo aha ntebekanisa yawe aha by’okwegaita omubiro by’omumaisho. Nikiha omuri ebi ekiri kubasa kushobororeraho kurungi entebekanisa zawe?

|  | Nyetebekanise kurinda mpaka nshwirwe ninga ntasize |
| --- | --- |
|  | Nyetebekanise kurinda mpaka mbwine aranshwere/ntahisye |
|  | Nyetebekanise kurinda mpaka mbwine omunntu wundikukunda |
|  | Nyetebekannise kwegaita natunga omugisha. |

**Ekicweka kya 7: okuza omurukundo omurundi gw’okubanza. (**yaba omukundwa owoyine hati niwe wabandise kwegaita nawe, hendereraho ebibuzo)

Hati nyine ebibuzo ebikwatiraine nobuwayegaita omurundi gw’okubanza.

1. Okaba oine emyaka engahi?………
2. Ogwe muntu aine emyaka engahi? Buririza aha myaka eyi aine obwahati …………………
3. Hakabaho emyezi ninga emyaka engahi kuruga watandika rukundo nogwo muntu nan’obu wabanza kwegaita nawe? .........................................
4. Obwire obu wabire ori omuri rukundo n’omuntu ogwe, haine ondijo owuwabire noreba/oinire rukundo?

|  | Ego |  | Ngaha |
| --- | --- | --- | --- |

1. Aha murundi gw’okubanza ogwe, eiwe ninga ogwe muntu mukakozesa omuringo gw’okubaririra oruzaro?

|  | Ego |  | Ngaha (guruka oze 67) |
| --- | --- | --- | --- |

1. Yaba eri ego, niguha?

|  | Kondomu |  | Obujuma |  | Ekikatu |  | Obwahakono |  | Omushaija kumarira aheru |  | Obutegaita |  | Ogwo kwekigira enda waheza  kwegaita oterinzire |
| --- | --- | --- | --- | --- | --- | --- | --- | --- | --- | --- | --- | --- | --- |

1. Okakwatwaho kugira ngu nobasa kutunga akoko kasirimu/sirimu ninga endijo ndwara ez’obushambani?

|  | Nkakwatwaho munonga |  | Nkakwatwaho konka timunonga |  | Tindakwatsirweho nakakye. (mara interview) |
| --- | --- | --- | --- | --- | --- |

1. Haine ekiwakozire kwenda kukyendeza aha migisha y’okukwatwa endwara?

|  | Ego |  | Ngaha |
| --- | --- | --- | --- |

1. Yaba eri ego, okakora ki? (buririza)……………
