# Supplementary material for: Sexual and reproductive health knowledge and practices among youth with and without mental illness in Uganda: a comparative study
Source: Trop Med Health. 2022 Aug 2;50:51. doi: 10.1186/s41182-022-00444-1 (PMC9344651; doi:10.1186/s41182-022-00444-1)
Supplement: Supplementary file 2 — Additional file 2. English Questionnaire. [file 41182_2022_444_MOESM2_ESM.docx]

**English Questionnaire**

**Section 1**: Socioeconomic and Demographic characteristics

1. Sex or respondent:

|  | Male |  | Female |
| --- | --- | --- | --- |

1. Age of Respondent ……………………………..
2. What is the highest level of Education you have completed?

|  | None |  | primary |  | Secondary |  | tertiary |
| --- | --- | --- | --- | --- | --- | --- | --- |

**Section 2:** Sources of information on, and knowledge of reproductive health

1. What are your sources of information on **puberty** (the ways in which boys' and girls' bodies change during the teenage years and what sources do you prefer or think is most accurate? (tick option given for a, b and c)

| Options | 1. Source | 1. Preferred | 1. Most accurate |
| --- | --- | --- | --- |
| School |  |  |  |
| Magazines and newspapers |  |  |  |
| Movies |  |  |  |
| Radio |  |  |  |
| Social media (facebook, watsapp) |  |  |  |
| Health workers |  |  |  |
| Friends/peers |  |  |  |
| parents |  |  |  |
| Siblings |  |  |  |
| Other (write option given) |  |  |  |

1. What does a girl starting to menstruate mean to you?

……………………………………………………………………………………………

1. What does a boy starting to have wet dreams mean to you?

……………………………………………………………………………………………

1. A woman can get pregnant on the very first time that she has sexual intercourse.

|  | True |  | False |  | Don’t know/ not sure |
| --- | --- | --- | --- | --- | --- |

1. A woman stops growing after she has had sexual intercourse for the first time.

|  | True |  | False |  | Don’t know/ not sure |
| --- | --- | --- | --- | --- | --- |

1. Masturbation causes serious damage to health.

|  | True |  | False |  | Don’t know/ not sure |
| --- | --- | --- | --- | --- | --- |

1. A woman is most likely to get pregnant if she has sexual intercourse half way between her periods.

|  | True |  | False |  | Don’t know/ not sure |
| --- | --- | --- | --- | --- | --- |

**Section 3:** Knowledge and usage of contraceptive methods

1. Which Contraceptive types/methods do you know (tick all mentioned options).

|  | Condoms |  | pills |  | injectable |  | implants |  | withdrawal |  | Abstinence |  | Emergency Contraception |
| --- | --- | --- | --- | --- | --- | --- | --- | --- | --- | --- | --- | --- | --- |

1. nat does this mean??What options are you referring to? Which methods do you think is most suitable for young people?

|  | Condoms |  | pills |  | injectable |  | implants |  | withdrawal |  | Abstinence |  | Emergency Contraception |
| --- | --- | --- | --- | --- | --- | --- | --- | --- | --- | --- | --- | --- | --- |

1. Do you know where to get the above methods? (If No skip to next section)

|  | Yes |  | No |
| --- | --- | --- | --- |

1. If yes which places do you know? (tick all options given)

|  | Government H/F |  | Private H/F |  | Drug shop/Pharmacy |  | Supermarket/shop |  | Youth center |
| --- | --- | --- | --- | --- | --- | --- | --- | --- | --- |

**Section 4: Knowledge of HIV/AIDS and sexually transmitted diseases**

1. Have you heard of HIV or AIDS (*use local terms*)?

|  | Yes |  | No |
| --- | --- | --- | --- |

1. How does a person get HIV infection

………………………………………………………………………………………….

1. I am now going to read you some statements about HIV/AIDS. Please tell me whether you think the statement is true, or false, or whether you don't know.
2. It is possible to cure AIDS

|  | True |  | False |  | Don’t know/ not sure |
| --- | --- | --- | --- | --- | --- |

1. I can tell a person who has HIV infection by just looking at them?

|  | True |  | False |  | Don’t know/ not sure |
| --- | --- | --- | --- | --- | --- |

1. A person with HIV always looks emaciated or unhealthy in some way

|  | True |  | False |  | Don’t know/ not sure |
| --- | --- | --- | --- | --- | --- |

1. People can take a simple test to find out whether they have HIV

|  | True |  | False |  | Don’t know/ not sure |
| --- | --- | --- | --- | --- | --- |

1. What are the ways in which a young person can protect themselves from getting HIV

|  | Use of condoms |  | Having one faithful partner |  | Abstaining from sex |
| --- | --- | --- | --- | --- | --- |

1. Have you ever tested for HIV?

|  | Yes |  | No |
| --- | --- | --- | --- |

1. Do you think it is important to test yourself for HIV?

|  | Yes |  | N0 |  | Don’t know/ not sure |
| --- | --- | --- | --- | --- | --- |

1. Do you think it is important to know your sexual partner’s HIV status?

|  | Yes |  | No |  | Don’t know/ not sure |
| --- | --- | --- | --- | --- | --- |

1. Apart from HIV/AIDS, have you heard about any other diseases that men and women can catch by having sexual intercourse.

|  | Yes |  | **No (skip to QN 25)** |
| --- | --- | --- | --- |

1. If yes, can you please mention those that you know?

………………………………………………………………………………………………

1. What are the signs and symptoms of a sexually transmitted diseases in a man? (did the participant mention atleast one correct answer?)

|  | Yes |  | No |
| --- | --- | --- | --- |

1. What are the signs or symptoms of a sexually transmitted disease in a woman? (did the participant mention atleast one correct answer?)

|  | Yes |  | No |
| --- | --- | --- | --- |

1. If a friend of yours needed treatment for a sexually transmitted disease, where could he or she obtain such treatment? (Did they mention a correct place eg clinic, healthfacility, pharmay, drug shop)

|  | Yes |  | No |
| --- | --- | --- | --- |

**Section 5:** Current/most recent heterosexual relationship

1. Have you ever had a girl/ boy friend? By girl/boy friend, I mean someone to whom you were sexually or emotionally attracted and whom you 'dated' (*use local terms to specify going out together unaccompanied by other adults*)

|  | Yes |  | No **( *Skip to section 6*)** |
| --- | --- | --- | --- |

1. How many girl / boy friends have you had? .........................................(if 1, skip section 7)

Ask the following sequence of questions about CURRENT (MOST RECENT) girl / boyfriend

(*to be called “that person”*)

1. How old is your current boyfriend/girlfriend?....……………………………..
2. During the time you were/have been 'dating' that person did you 'date'/have you ‘dated’ anyone else?

|  | Yes |  | No |
| --- | --- | --- | --- |

1. How would you describe your relationship with that person? Was (is) it

|  | Casual friendship |
| --- | --- |
|  | Serious relationship but with no intention of marriage |
|  | An important relationship that might lead to marriage |

1. Did you and that person have any physical contact, such as holding hands or hugging?

|  | Yes |  | No |
| --- | --- | --- | --- |

1. Did you ever kiss that person on the lips?

|  | Yes |  | No |
| --- | --- | --- | --- |

1. Did you and that person ever touch each others private parts?

|  | Yes |  | No |
| --- | --- | --- | --- |

1. Did you and that person ever have sexual intercourse?

|  | Yes |  | No **(skip to section 6)** |
| --- | --- | --- | --- |

**Section for those who have ever had sexual intercourse**

1. Think back to the first time you had sexual intercourse with your current boyfriend/girlfriend, Would you say. READ OUT

|  | I forced that person to have intercourse against her/his will |
| --- | --- |
|  | I persuaded that person to have intercourse |
|  | That person persuaded me to have intercourse |
|  | That person forced me to have intercourse |
|  | We were both equally willing |

1. And would you say it was planned or unexpected?

|  | Planned |  | Unexpected |
| --- | --- | --- | --- |

1. Was this the first time that you had full sexual intercourse in your life? **(if yes skip section 7)**

|  | Yes |  | No |
| --- | --- | --- | --- |

1. How old were you at the time you first had sex with that person?......................................
2. On that first time did you or that person do anything to avoid a pregnancy?

|  | Yes |  | No (**skip to QN 43)** |
| --- | --- | --- | --- |

1. If yes, What method did you use?

|  | Condoms |  | pills |  | injectable |  | implants |  | withdrawal |  | Abstinence |  | Emergency Contraception |
| --- | --- | --- | --- | --- | --- | --- | --- | --- | --- | --- | --- | --- | --- |

1. Did you ever discuss contraception with that person? IF YES Did you discuss contraception before or after you first had intercourse?

|  | Never |  | Before sexual intercourse |  | After sexual intercourse |
| --- | --- | --- | --- | --- | --- |

1. Apart from the first time, did you and that person ever use other methods to avoid pregnancy?

|  | Yes always |  | Yes sometimes |  | Never **(skip to Qn 46)** |
| --- | --- | --- | --- | --- | --- |

1. What methods did you and that person mostly use?

|  | Condoms |  | pills |  | injectable |  | implants |  | withdrawal |  | Abstinence |  | Emergency Contraception |
| --- | --- | --- | --- | --- | --- | --- | --- | --- | --- | --- | --- | --- | --- |

1. Were you ever concerned that you might catch AIDS or another sexually transmitted disease from THAT PERSON? IF YES, how concerned were you?

|  | Very concerned |  | Somewhat concerned |  | Not concerned **(skip to section 6)** |
| --- | --- | --- | --- | --- | --- |

1. Were you able to do anything to reduce the risk of infection

|  | Yes |  | No |
| --- | --- | --- | --- |

1. If yes, What did you do? *Probe*

…………………………………………………………………………..

**Section 6:** Types of heterosexual contact

*Opening statement if participant had not had a girl/boyfriend before:*

You told me that you have had no girl/boyfriends. I now want to ask you about any sexual contacts that you may have experienced.

*Opening statement for participants with or have had a boy/girlfriend before:*

You have told me about your relationship with THAT PERSON. Apart from her/him and any earlier girl /boyfriends, I now want to ask you about other types of sexual partners that you may have experienced

1. Some young people have 'one night stands' (*use local terms*), perhaps after a party or after drinking? Has this ever happened to you?

|  | Yes |  | No **(skip to QN 53)** |
| --- | --- | --- | --- |

1. How many 'one night stands' have you had?..............................................................
2. Did you or the sexual partner use any contraceptive methods on these “one night stands”?

|  | Always |  | Sometimes |  | Never |
| --- | --- | --- | --- | --- | --- |

1. If always or sometimes, which contraceptive methods were used?

|  | Condoms |  | pills |  | injectable |  | implants |  | withdrawal |  | Abstinence |  | Emergency Contraception |
| --- | --- | --- | --- | --- | --- | --- | --- | --- | --- | --- | --- | --- | --- |

1. Some young people pay money or gifts in exchange for sexual intercourse. Has this ever happened to you?

|  | Yes |  | No |
| --- | --- | --- | --- |

1. Some people receive money or gifts in exchange for sexual intercourse. Has this ever happened to you? (If no skip to 64).

|  | Yes |  | No |
| --- | --- | --- | --- |

1. If yes; How many women/men have you had sex with for money or gifts?......………
2. Did you or the sexual partner do anything to avoid a pregancy on these occasions?

|  | Always |  | Sometimes |  | Never |
| --- | --- | --- | --- | --- | --- |

1. If always or sometimes, which contraceptive methods were used?

|  | Condoms |  | pills |  | injectable |  | implants |  | withdrawal |  | Abstinence |  | Emergency Contraception |
| --- | --- | --- | --- | --- | --- | --- | --- | --- | --- | --- | --- | --- | --- |

1. In your whole life how many people have you had sexual intercourse with ...........................
2. **FOR THOSE WHO HAVE NEVER EXPERIENCED SEXUAL INTERCOURSE**

| 59 | People may have mixed reasons for not having intercourse. I will read out some reasons. Please tell me for each reason whether it applies to you or not. | Agree | Disagree | Don’t know/not sure |
| --- | --- | --- | --- | --- |
| 1 | I don't feel ready to have sex. |  |  |  |
| 2 | I have not had the opportunity. |  |  |  |
| 3 | I think that sex before marriage is wrong |  |  |  |
| 4 | I am afraid of getting pregnant or impregnating a girl |  |  |  |
| 5 | I am afraid of getting HIV/AIDS or another sexually transmitted infection. |  |  |  |

1. And now I have a question about your future plans about sexual intercourse. Which of these statement best describes your plans?

|  | I plan to wait until marriage |
| --- | --- |
|  | I plan to wait until I am engaged to be married |
|  | I plan to wait until I find someone I love |
|  | I plan to have sexual intercourse when an opportunity comes along |

**Section 7:First sexual relationship (If current boy/girlfriend is first sexual partner, end the interview).**

Now I have some question about the first time that you had sexual intercourse.

1. How old were you at that time?………
2. How old was that person at that time? Probe for current age …………………
3. How many months or years were there between the time you started your relationship and the time you first had sex with that person? ....................................................
4. And would you say the sex was planned or unexpected?

|  | Planned |  | Unexpected |
| --- | --- | --- | --- |

1. On that first time did you or that person use a contraceptive method?

|  | Yes |  | No **(skip to Qn 67)** |
| --- | --- | --- | --- |

1. If yes, what method did you use?

|  | Condoms |  | pills |  | injectable |  | implants |  | withdrawal |  | Abstinence |  | Emergency Contraception |
| --- | --- | --- | --- | --- | --- | --- | --- | --- | --- | --- | --- | --- | --- |

1. Did you ever discuss contraception with that person? IF YES Did you discuss contraception before or after you first had intercourse?

|  | Before first intercourse |  | After first intercourse |  | Never |
| --- | --- | --- | --- | --- | --- |

1. Were you ever concerned that you might catch AIDS or another sexually transmitted disease from that person?

|  | Very concerned |  | Somewhat concerned |  | Not concerned (end interview) |
| --- | --- | --- | --- | --- | --- |

1. Did you do anything to reduce the risk of infection?

|  | Yes |  | No |
| --- | --- | --- | --- |

1. If yes, what did you do? (Probe)……………………………………………………

Appendix 4: Runyankore Questionnaire

**Ekicweka kya 1**: Ebiri kukukwataho nana kworikuhikana n’abantu hamwe n’entasya Socioeconomic and Demographic characteristics

1. Obuhangwa bw’owayetaba omu okucondoza

|  | Omushaija/omutsigazi |  | Omwishiki/omukazi |
| --- | --- | --- | --- |

1. Emyaka y’owayetaba omu kucondoza ……………………………..
2. Okashoma wahika omu kyakangahi?

|  | Tindashomire/Tahariho |  | Purimare |  | Siniya |  | Tekiniko/kolegi |
| --- | --- | --- | --- | --- | --- | --- | --- |

**Ekicweka kya 2:** ahi ori kwiha okumanyisibwa nan’obwengye ahaby’amagara ebikwateirine nan’oruzaro/eby’okuzara/okwegaita.

1. Notunga ota okumanyisibwa haby’omushogoyo? kuruga nkahi(egyero eyi emibiri yabojo n’abaishiki eri kuhinduka baba bari omu myaka y’okunyeta/y’obunyeto kandi ni bukomoko ki obu ori kwesiga ninga obwori kuteekateeka ngu nibwo buhikire kimwe?) (gorora ekisharamo kimwe ahabya hebwoyo habwa a, b hamwe na c)

| ekyokusharamu | 1. Obukomoko | 1. Ekirikukirayo preferred | 1. Obuhikire kimwe |
| --- | --- | --- | --- |
| Ah’ishomero |  |  |  |
| Zamagazinis hamwe n’amahurire |  |  |  |
| Emizano/zafirimu |  |  |  |
| Radiyo |  |  |  |
| Ahamikutu yokuhikanisa abantu (facebook, watsapp) |  |  |  |
| Abashaho |  |  |  |
| Emikago/abanywani |  |  |  |
| Abazaire |  |  |  |
| Abinkuzarwa nabo |  |  |  |
| Ahandi(handika omuhanda ogu waha) |  |  |  |

1. Omwishiki kutandika kuza omumicwe/omukwezi nikimanyisa ki ahar’iwe/nokikyenga ota?

……………………………………………………………………………………………

1. Omwojo okunyama akarota haza akashohoza amaizi g’ekishaija atakimanyise nikimanyisa ki ahar’iwe?

……………………………………………………………………………………………

1. Omukazi nabasa kutwara enda omurundi gwe gwokubaza kwegaita/kuterana n’omushaija.

|  | Namazima |  | Timazima |  | Tinkumanya/tinkineho buhame |
| --- | --- | --- | --- | --- | --- |

1. Omukazi narekyeraho kukura yaheza kuterena/kwegaita n’omushaija omurundi gwokubanza.

|  | Namazima |  | Timazima |  | Ttikumanya/Tinkineho buhame |
| --- | --- | --- | --- | --- | --- |

1. Kuzanisa ebicweka byawe by’ekihama kweshemeza kine akabi kahahango ah’amagara.

|  | Namazima |  | Timazima |  | Ttikumanya/tinkineho buhame |
| --- | --- | --- | --- | --- | --- |

1. Omukazi nabasa kutwara enda yayegaita/yaterana n’omushaija rwagati yo kwezi kwe micwe.

|  | Namazima |  | Timazima |  | Tikumanya/tinkineho buhame |
| --- | --- | --- | --- | --- | --- |

**Ekicweka kya 3:** Okumanya hamwe n’enkozesa y’emiringo y’embaririra yaruzaro

1. Emiringo y’okubarira oruzaro ey’orikmanya neha(tikinga eyagambwaho)

|  | kodomu/obupira |  | Obujuma |  | Ekikato |  | obwomumukono |  | Omushaija kumarira aheru |  | Obutegaita n’omushaija |  | Ogwo kwekigira enda waheza  kwegaita oterinzire |
| --- | --- | --- | --- | --- | --- | --- | --- | --- | --- | --- | --- | --- | --- |

1. nat does this mean??What options are you referring to?Notekateka ngu emuringo eshemerire eminyeto neha

|  | kodomu/obupira |  | Obujuma |  | Ekikato |  | obwomumukono |  | Omushaija kumarira aheru |  | Obutegaita n’omushaija |  | Ogwo kwekigira enda waheza  kwegaita oterinzire |
| --- | --- | --- | --- | --- | --- | --- | --- | --- | --- | --- | --- | --- | --- |

1. Nomanya aho kwiha emiringo eyagambwaho haruguru?

|  | Ego |  | Ngaha |
| --- | --- | --- | --- |

1. Yaba ego, nemyanya eha eyorikumany?(tikinga emiringo eya hebwa)

|  | Omw’irwariro rya gavumenta |  | Omw’irwariro ry’omuntu buntu |  | Eduuka y’emibazi |  | Supamaketi/eduuka |  | Ahi eminyeto eri kwerundanira. |
| --- | --- | --- | --- | --- | --- | --- | --- | --- | --- |

**Ekicweka kya 4 Eki ori kumanya aha kakoko kasirimu/sirimu hamwe n’endwara z’obushambani.**

1. Warahurireho aha kakoko ka sirimu ninga sirimu (*use local terms*)?

|  | ego |  | ngaha |
| --- | --- | --- | --- |

1. Omuntu natunga ata akakoko ka sirimu?

………………………………………………………………………………………….

1. Hati naza kukushomera bimwe habikwatairine n’akakoko ka sirimu/sirimu. Ngambira yaba namazima, ninga timazima ninga torikumanya
2. Nikibasika kukiza sirimu

|  | Namazima |  | Timazima |  | Tinkumanya/tikineho buhame |
| --- | --- | --- | --- | --- | --- |

1. Nimbasa kureba omuntu ngambe yaba aine akakoko kasirimu/Nimbasa kumanya omuntu yaba ayine akakoko kasirimu namureba bureba.

|  | Anamazima |  | Timazima |  | Tikumanya/tikineho buhame |
| --- | --- | --- | --- | --- | --- |

1. Omuntu aine akakoko kasirimu naba ahweire amagufa ninga naba aine amagara mabi omumuringo gutari gumwe.

|  | Namazima |  | Timazima |  | Tikumanya/tikineho buhame |
| --- | --- | --- | --- | --- | --- |

1. Abantu nibabasa kuceberwa omumuringo gworobi/gwanguhi munonga kumanya yaba baine akakoko kasirimu

|  | Namazima |  | Timazima |  | Tikumanya/tikineho buhame |
| --- | --- | --- | --- | --- | --- |

1. Ningyero ki eyi omunyeto gukubasa kwekumamo bbutatunga akakoko kasirimu.

|  | Kukozesa kondomu |  | Kugira omukundwa omwe omwesigwa |  | Obutashambana/obutegaita |
| --- | --- | --- | --- | --- | --- |

1. Wara yekyebizeho akakoko kasirimu?

|  | ego |  | Ngaha |
| --- | --- | --- | --- |

1. Noteekateeka ngu nikikuru kwekyebeza akakoko kasirimu

|  | Ego |  | Ngaha |  |  |
| --- | --- | --- | --- | --- | --- |

1. Notekateka nikikuru kumanya omukundwa wawe kwayemerire omubya kakoko kasirimu?

|  | Ego |  | Ngaha |  |  |
| --- | --- | --- | --- | --- | --- |

1. . Oihireho akakoko kasirimu/sirimu warahurireho endijo ndwara ei abashaija ninga abakazi bakukwatwa ahanyima yokushambana?

|  | Ego |  | Ngaha ( guruka oze 25) |
| --- | --- | --- | --- |

1. Yaba ego, gamba ezo ezorikumanya?

………………………………………………………………………………………………

1. Obubonero bw’endwara z’obushambani omubashaija nibuha?

…………………………………………………………………………………………………

1. Obubonero bw’endwara z’obushambani omu bakazi nibuha?

………………………………………………………………………………………………….

1. Munywani wawe kuyakuba nayetaga obujanjabi bw’endwara y’obushambani, nabwiha hi?

………………………………………………………………………………………………………

.

**Ekicweka kya 5:** okuza omumubonano/okwegaita obwahati/obu herurukire

1. Waragizireho omukundwa/omurigirwa? Omukundwa/omurigirwa nimanyisa omwojo/omwishiki ouwahurire wakunda munonga ninga kwenda kwegaita nawe kandi ouwagyenzire nawe awutu/kutambaramu hataine muntu mukuru owumugyenzire nawe?.

|  | Ego |  | Ngaha( Guruka oze kicweka 6) |
| --- | --- | --- | --- |

1. Ogizire abakundwa/abarigirwa bangahi?...............................(yaba omwe, guruka kicweka 7)

Buza ebibuzo ebyakurataho bikwatiraine aha bwahati(omwahihi/omubiro bitari byahare) omukundwa/omurigirwa(mwete “omuntu ogwe”)

1. Omuntu ogwe aine emyaka engahi? Buuza emyaka ye hati? …………………………..
2. Omubwire obwo obu wabire ori/obu obire omuri rukundo n’omuntu ogwe, obire wagizire rukundo nomuntu ondijo?

|  | Ego |  | Ngaha |
| --- | --- | --- | --- |

1. Nobasa kushoborore ota omukago gwawe nogwo muntu? Gukaba (nogwo)

|  | Omunywani bunywani/omunywani kyonka. |
| --- | --- |
|  | Nomukago guhami konka gutaine ekigyendererwa kyo kushwerana/Okutasya. |
|  | Omukago mukuru gukubasa kuhendera omubushwere/okutasya. |

1. Eiwe nogwo muntu mukatunga okukwatana kwe mibiri yanyu, tugire nga okwekwata omugaro, okwefumbata omukifuba/okwegwa omunnda ninga okwenywegyera.

|  | Ego |  | Ngaha |
| --- | --- | --- | --- |

1. Okanywegyeraho ogwe muntu aha minywa?

|  | Ego |  | Ngaha |
| --- | --- | --- | --- |

1. Eiwe n’ogwo muntu mwarayekwasireho omubicweka by’ekihama?

|  | Ego |  | Ngaha |
| --- | --- | --- | --- |

1. Eiwe nogwo muntu mwara yegaisireho?

|  | Ego |  | Ngaha (guruka oze kicweka 6) |
| --- | --- | --- | --- |

**Ekicweka kyabo abarashambaineho/abarayegaisireho.**

1. Teekateeka enyimaho obu wayegaita nomukundwa wawe owoyine obwahati omurundi gwokubanza, nogira ngu. SHOMA EBI.

|  | Nkagyema omuntu ogwe kwegaita atarikwenda |
| --- | --- |
|  | Nkabihabiha omuntu ogwe kwegaita |
|  | Omuntu ogwe akambihabiha kwegaita |
|  | Omuntu ogwwe akagyema kwegaita |
|  | Twena/twembiri tukaba nitukyenda |

1. Kandi nobasa kugira ngu kikaba kiteebeekanisibwe ninga kitateekateekirwe?

|  | Kitebekanisibwe |  | Kitateekateekirwe |
| --- | --- | --- | --- |

1. Ogu nigwo gwabire guri omurundi gwawe gwokubanza kwegaita kuhika aha mbugiro omugara gawe?

|  | Ego |  | Ngaha |
| --- | --- | --- | --- |

1. Okaba oine emyaka engahi obu wayegaita n’omuntu ogwe omurunudi gwokubanza?.....................................
2. Aha murundi gw’okubanza ogwe, eiwe ninga ogwe muntu haine ayakozire ekintu kyona kukingira/kwerinda kutwara enda?

|  | Ego |  | Ngaha (guruka oze 43) |
| --- | --- | --- | --- |

1. Yaba ego, nomuringo guha ogu wakozise?.....................................................................

|  | Kondomu |  | Obujauma |  | Ekikatu |  | Obwahamukono |  | Omushaijakumariraaheru |  | Obutegaita |  | Ogwo kwekigira enda waheza  kwegaita oterinzire |
| --- | --- | --- | --- | --- | --- | --- | --- | --- | --- | --- | --- | --- | --- |

1. Waraganiraho aha by’okubaririra oruzaro n’omuntu ogwe? Yaba ego okaganira aha by’okubaririra oruzaro mutakegisire ninga mwaherize kwegaita omurundi gw,okubanza?

|  | Tindakiganireho |  | Tutakegaisire |  | Hanyuma y’okwegaita. |
| --- | --- | --- | --- | --- | --- |

1. Oihireho omurundi gwokubanza, eiwe nan’ogwe muntu mwara koziseho omuringo ogundi kwerinda kutwara enda?

|  | Ego burikaire |  | Ego obumwe |  | Tikikabahoga (guruka oze 46) |
| --- | --- | --- | --- | --- | --- |

1. Eiwe n’omuntu ogwe nimukira kukozesa muringo ki?

|  | Kondomu/obupira |  | Obujuma |  | Ekikatu |  | Obwahamukono |  | Omushaija kamarira aheru |  | Obutagaita |  | Ogwo kwekigira enda waheza  kwegaita oterinzire/otekumire |
| --- | --- | --- | --- | --- | --- | --- | --- | --- | --- | --- | --- | --- | --- |

1. Wara gizire okukwatwaho kugira ngu nobasa kukwatwa sirimu ninga endijo endwara y’obushambani kuruga aha muntu ogwe? Yaba ego, okakwatwoho ekirikwingana ki?

|  | Nkakwatwaho munonga |  | nkakwataho kukye |  | Tidakwatsirweho (guruka oze kicweka 6) |
| --- | --- | --- | --- | --- | --- |

1. Okabasa kugira eki wakora kucendeza aha migisha y’okukwatwa endwara?

|  | Ego |  | Ngaha |
| --- | --- | --- | --- |

1. Yaba eri ego, okakora ki? Buririza?…………………………………………………………………………..

**Ekicweka 6:** ebika by’okwegaita nabakazi/abashaija.

*Opening statement if participant had not had a girl/boyfriend before:*

Waba wangambire ngu tokagira omurigirwa/omukundwa. Hati ninyenda kukubuza aha miriingo yokwegaita yona eyi wara rabiremu.

*Opening statement for participants with or have had a boy/girlfriend before:*

Waba wangambire akakwate kwawe n’omuntu ogwe. Oihireeho we hamwe nomukundwa wena eyabandize, hati nenyenda kukubuza aha bika bya bakundwa abandi abu wakubasa kuba warabiremu.

1. Eminyeto emwe/abana bato nibegaita rumwe na rumwe batakimanyise/bata kyetebekanisise, katugire hanyuma y’embaga ninga baheza kunywamu. Eki kyarakubireho?

|  | Ego |  | Ngaha (guruka oze 58) |
| --- | --- | --- | --- |

1. Oyegaisire emirundi engahi erya rimwe na rimwe otakyetebekanisise?..................................
2. Eiwe ninga omukundwa wawe akakozesa omuringo gwona gw’okubariri oruzaro obumwayegaita muteeteebekanise?

|  | Buri kaire |  | Obumwe |  | Tikirabireho **(skip 52**) |
| --- | --- | --- | --- | --- | --- |

1. Yaba buri kaire ninga obumwe, nimuringo ki ogu mwakozise?

|  | Kondomu/obupira |  | Obujuma |  | Ekikato |  | Akahamukono |  | Omushaija kumarira aheru |  | Obutegaita |  | Ogwo kwekigira enda waheza  kwegaita oterinzire |
| --- | --- | --- | --- | --- | --- | --- | --- | --- | --- | --- | --- | --- | --- |

1. Eminyeto emwe/abana bato nibashashura sente ninga ebihembo kwenda kwegaita?eki kyarakubireho?

|  | Ego |  | Ngaha |
| --- | --- | --- | --- |

1. Eminyeto emwe/abana nibakira/nihebwa esente nninga ebihembo kwenda kwegaita. Eki kyarakubireho?

|  | Ego |  | Ngaha |
| --- | --- | --- | --- |

1. Yaba ego, n’abashaija/abakazi bangahi abu oyegaisire nabo habwe sente ninga ebihembo? …………………………………………………………………………..
2. Eiwe ninga ouwayegaisire nawe haine eyakozire ekintu kyona kwenda kwerinda enda obwire bwe?

|  | Buri kaire |  | Obumwe |  | Tikirabireho |
| --- | --- | --- | --- | --- | --- |

1. Yaba buri kaire ninga obumwe, nimuringo ki gw’embaariri yaruzaro ogu mwakozise?

|  | Kondomu/obupira |  | Obujuma |  | Ekikato |  | Akahamukukono |  | Omushaija kumarira aheru |  | Obutegaita |  | Ogwo kwekigira enda waheza  kwegaita oterinzire |
| --- | --- | --- | --- | --- | --- | --- | --- | --- | --- | --- | --- | --- | --- |

1. Omu magara gawe gona oyegaisire nabantu bangahi? …......................................
2. NEBY’ABO ABATAKEGAITAGA

| 59 | Abantu nibabasa kugira enshonga zitarikushana habwenki batarikwegaita. Ninza kukushomera zimwe aha shonga. Ninshaba ongambire buri hashonga yaba nekukwataho ninga terikukwataho. | Ninyikirizana nayo | Tikwikirizana nayo | Tindikumany/tikineho buhame. |
| --- | --- | --- | --- | --- |
| 1 | Nimpurira ntaketeeketeekire kwegaita. |  |  |  |
| 2 | Tinkatungire mugisha |  |  |  |
| 3 | Niteekateeka ngu akwegaita ntankashwire kigwire. |  |  |  |
| 4 | Nintina kutwara enda /okutweka |  |  |  |
| 5 | Nintina kukwatwa akakoko kasirimu/sirimu ninga ezinda endwar z’obushambani. |  |  |  |

1. Kandi hati nyine ekibuzo aha ntebekanisa yawe aha by’okwegaita omubiro by’omumaisho. Nikiha omuri ebi ekiri kubasa kushobororeraho kurungi entebekanisa zawe?

|  | Nyetebekanise kurinda mpaka nshwirwe ninga ntasize |
| --- | --- |
|  | Nyetebekanise kurinda mpaka mbwine aranshwere/ntahisye |
|  | Nyetebekanise kurinda mpaka mbwine omunntu wundikukunda |
|  | Nyetebekannise kwegaita natunga omugisha. |

**Ekicweka kya 7: okuza omurukundo omurundi gw’okubanza. (**yaba omukundwa owoyine hati niwe wabandise kwegaita nawe, hendereraho ebibuzo)

Hati nyine ebibuzo ebikwatiraine nobuwayegaita omurundi gw’okubanza.

1. Okaba oine emyaka engahi?………
2. Ogwe muntu aine emyaka engahi? Buririza aha myaka eyi aine obwahati …………………
3. Hakabaho emyezi ninga emyaka engahi kuruga watandika rukundo nogwo muntu nan’obu wabanza kwegaita nawe? .........................................
4. Obwire obu wabire ori omuri rukundo n’omuntu ogwe, haine ondijo owuwabire noreba/oinire rukundo?

|  | Ego |  | Ngaha |
| --- | --- | --- | --- |

1. Aha murundi gw’okubanza ogwe, eiwe ninga ogwe muntu mukakozesa omuringo gw’okubaririra oruzaro?

|  | Ego |  | Ngaha (guruka oze 67) |
| --- | --- | --- | --- |

1. Yaba eri ego, niguha?

|  | Kondomu |  | Obujuma |  | Ekikatu |  | Obwahakono |  | Omushaija kumarira aheru |  | Obutegaita |  | Ogwo kwekigira enda waheza  kwegaita oterinzire |
| --- | --- | --- | --- | --- | --- | --- | --- | --- | --- | --- | --- | --- | --- |

1. Okakwatwaho kugira ngu nobasa kutunga akoko kasirimu/sirimu ninga endijo ndwara ez’obushambani?

|  | Nkakwatwaho munonga |  | Nkakwatwaho konka timunonga |  | Tindakwatsirweho nakakye. (mara interview) |
| --- | --- | --- | --- | --- | --- |

1. Haine ekiwakozire kwenda kukyendeza aha migisha y’okukwatwa endwara?

|  | Ego |  | Ngaha |
| --- | --- | --- | --- |

1. Yaba eri ego, okakora ki? (buririza)……………
